# Supplementary material for: Integrative Metabolomic and Transcriptomic Analysis Elucidates That the Mechanism of Phytohormones Regulates Floral Bud Development in Alfalfa
Source: Plants (Basel). 2024 Apr 11;13(8):1078. doi: 10.3390/plants13081078 (PMC11053841; doi:10.3390/plants13081078)
Supplement: Supplementary file 1 [file plants-13-01078-s001.zip › Supplemental Figures - Floral bud.docx]

**Supplemental Figures**


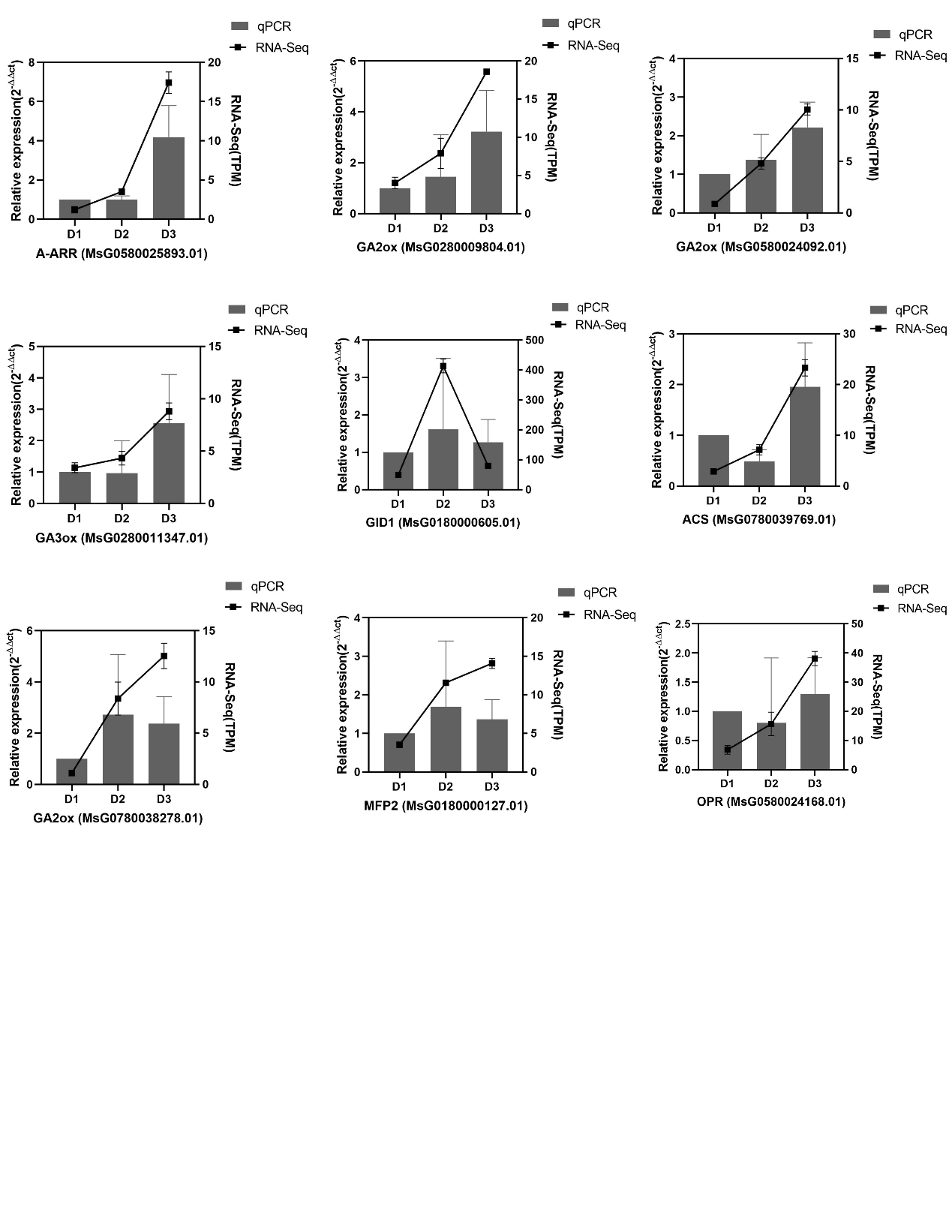


**Supplemental Figure S1: Verification of nine DEGs by qRT-PCR. Data are the means of three replicates.**

**
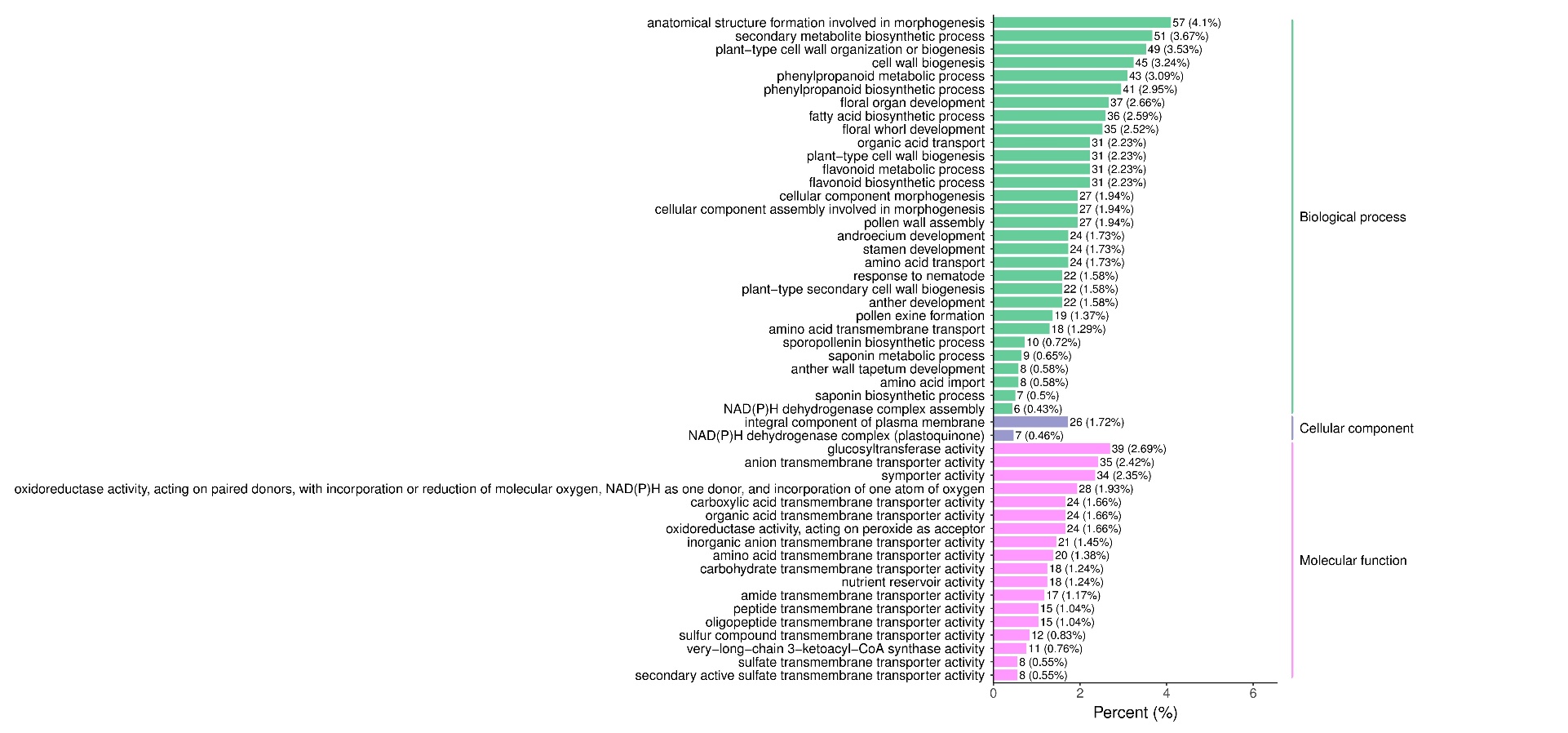

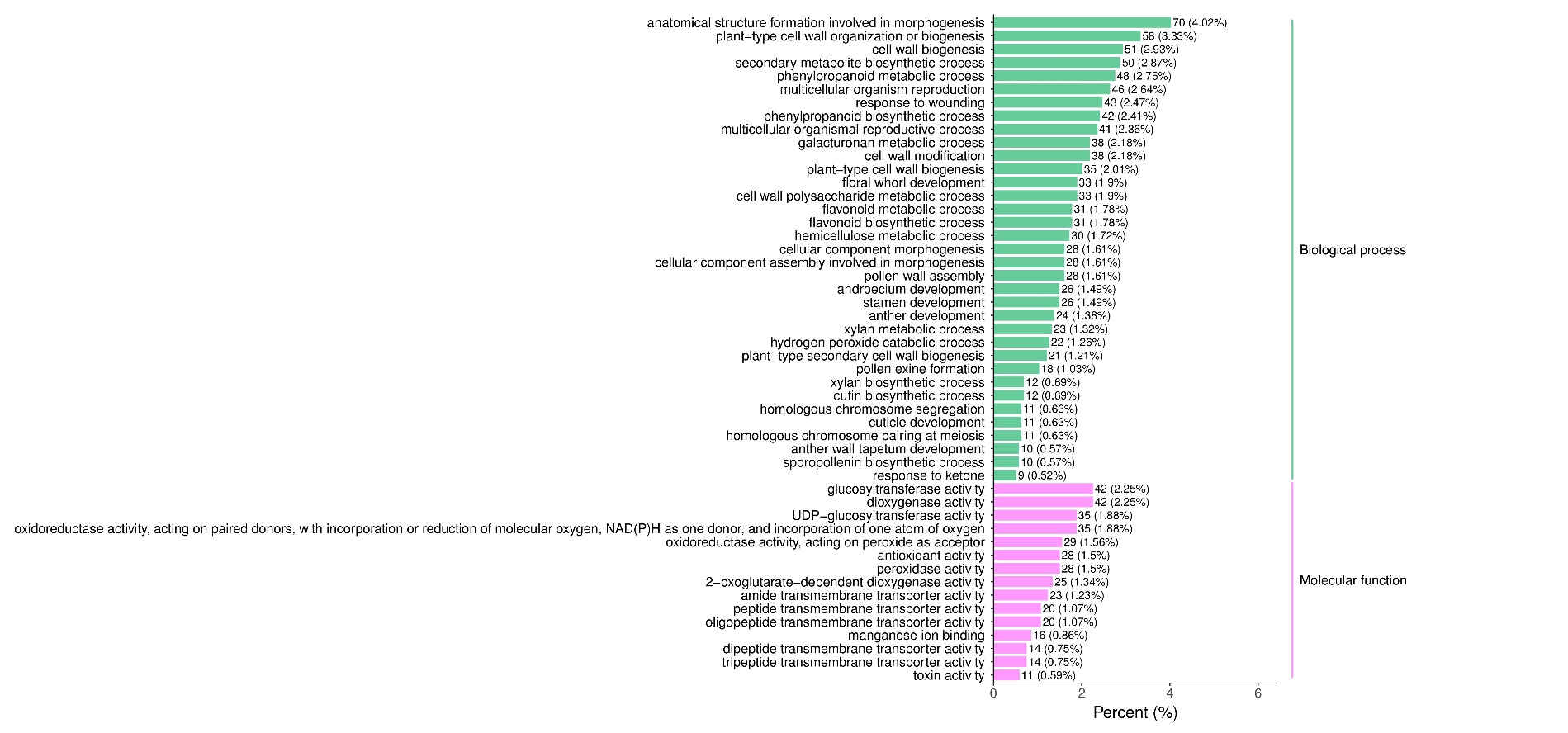
Supplemental Figure S2: GO enrichment analysis in the D2 vs. D1 and D3 vs. D2 comparison.**
